# Association between sarcopenia, physical performance and falls in patients with rheumatoid arthritis: a 1-year prospective study

**DOI:** 10.1186/s12891-021-04605-x

**Published:** 2021-10-18

**Authors:** Sabine Wiegmann, Gabriele Armbrecht, Diana Borucki, Bjoern Buehring, Frank Buttgereit, Christian Detzer, Désirée Schaumburg, Kim Nikola Zeiner, Roswitha Dietzel

**Affiliations:** 1 Charité – Universitätsmedizin Berlin, corporate member of Freie Universität Berlin and Humboldt- Universität zu Berlin, Klinik für Radiologie, Zentrum für Muskel- und Knochenforschung, Hindenburgdamm 30, 12200 Berlin, Germany; 2 Deutsche Rheuma-Liga Bundesverband e.V, Welschnonnenstraße 7, 53111 Bonn, Germany; 3 Rheumazentrum Ruhrgebiet, Ruhr-Universität-Bochum, Claudiusstr. 45, 44649 Herne, Germany; 4 Charité – Universitätsmedizin Berlin, corporate member of Freie Universität Berlin and Humboldt- Universität zu Berlin, Medizinische Klinik mit Schwerpunkt Rheumatologie u. Klinische Immunologie, Charitéplatz 1, 10117 Berlin, Germany; 5 Department of Dermatology, Venereology and Allergology, Goethe University Frankfurt, Theodor-Stern-Kai 7, 60596 Frankfurt/Main, Germany

**Keywords:** Rheumatoid arthritis, Fall, Risk factor, Balance, HAQ, Physical performance, Sarcopenia, FICSIT, Mechanography, SPPB

## Abstract

**Background:**

Patients with rheumatoid arthritis (RA) are at increased risk of falls and fractures. Sarcopenia occurs more frequently in RA patients due to the inflammatory processes. Early diagnosis and prevention programmes are essential to avoid serious complications. The present study aims to identify risk factors for falls related to sarcopenia and physical performance.

**Methods:**

In a 1-year prospective study, a total of 289 patients with RA, ages 24–85 years, were followed using quarterly fall diaries to report falls. At the baseline, medical data such as RA disease duration and Disease Activity Score (DAS28_CRP_) were collected. Self-reported disability was assessed using the Health Assessment Questionnaire (HAQ). Appendicular skeletal mass was determined by Dual X-ray-Absorptiometry (DXA). Physical performance was evaluated by handgrip strength, gait speed, chair rise test, Short Physical Performance Battery, and FICSIT-4. Muscle mechanography was measured with the Leonardo Mechanograph®. Sarcopenia was assessed according to established definitions by the European Working Group on Sarcopenia in Older People (EWGSOP2) and The Foundation for the National Institutes of Health (FNIH). Univariate and multiple logistic regression analysis were used to explore associations with falling**.** Receiver-operating characteristics (ROC) were performed, and the area under the curve is reported.

**Results:**

A total of 238 subjects with RA completed the 1-year follow-up, 48 (20.2%) experienced at least one fall during the observational period. No association was found between sarcopenia and prospective falls. Age (OR = 1.04, CI 1.01–1.07), HAQ (OR = 1.62, 1.1–2.38), and low FICSIT-4 score (OR = 2.38, 1.13–5.0) showed significant associations with falls.

**Conclusions:**

In clinical practice, a fall assessment including age, self-reported activities of daily life and a physical performance measure can identify RA patients at risk of falling.

**Trial registration:**

The study has been registered at the German Clinical Trials Register and the WHO International Clinical Trials Registry Platform (ICTRP) since 16 March 2017 (DRKS00011873).

## Key points

- Patients with rheumatoid arthritis have an increased risk of falls
- The association between sarcopenia and falls could not be found in a sample of patients with rheumatoid arthritis
- Age, HAQ, and low score of FICSIT-4 were associated with prospective falls
- Sex was not found to be a significant risk factor for falls
- An association between muscle power and prospective falls was not found
- A fall assessment including age, self-reported activities of daily life and a physical performance measure can identify RA patients at risk of falling

## Background

Rheumatoid arthritis (RA) is a complex autoimmune disease with a broad spectrum of manifestations influencing activities of daily life and physical performance. The prevalence of RA has been identified in a global study as one of the highest for Western Europe, causing a high burden on regional healthcare systems [1]. The reported diagnostic prevalence for Germany is 1.08% with increasing tendency [2]. For women, a 2.5-fold higher prevalence has been described than for men (1.49% vs. 0.62%) [2]. The peak incidence rate occurred in high-income countries in the age group between 65 and 79 years [2].

Subjects with RA have an increased risk for falls compared to healthy subjects [3–5]. The reported incidence of falls in populations with RA ranges between 36 and 50% in prospective studies [4–7], while in populations of healthy older individuals the fall incidence ranges between 6 and 34% [8]. In general, falls have a high impact on health care systems as fall-related injuries are associated with higher mortality and morbidity. Compared to 22 other European countries, the burden of falls in Germany is ranked 8th in terms of fall incidence and 10th in disability-adjusted life year (DALY) [9].

Reduced muscle strength in combination with low muscle mass and low physical performance is defined as sarcopenia [10]. The European Sarcopenia Working Group on Sarcopenia in Older People (EWGSOP) described two types of sarcopenia: ‘primary’ refers to age-related sarcopenia and ‘secondary’ is caused by various factors, including systemic diseases and inflammatory processes such as in rheumatoid arthritis [10]. The prevalence of sarcopenia is higher in patients with RA than in the general population [11]. Furthermore, sarcopenia is discussed to increase the risk of falls [17, 11].

Rheumatic disease-specific risk factors for falls can be classified as physiological, pharmacological, extrinsic and measure of disease activity [5]. In particular, structural changes such as joint disorders (pain, swollen joints) [12] and deformities [13], reduced postural control [14] and reduced muscle strength [15] were identified as potential physiological risk factors. The Disease Activity Score (DAS28), a clinical measure for disease activity, is also associated with falls [15]. Evidence exists that high scores in the Health assessment questionnaire (HAQ) are associated with falls [12, 15]. Aged over 65 years, the female sex and low BMI are evaluated as general risk factors for falls, yet not clearly confirmed in individuals with RA [5, 9].

In recent years, mechanography-based assessments have expanded the investigation of muscle strength, muscle power, and physical performance in various study populations. There is some evidence for associations of jumping mechanography with falls [16, 17]. There is little published data on mechanography assessments in patients with RA or on predicting falls in this population [18, 19].

Due to an increased risk of fracture in patients with RA [20, 21] including a higher risk for hip fractures [21], the impact of rheumatoid arthritis-associated falls should not be underestimated and their risks should be identified early with a focus on prevention. Therefore, the primary objective of this study was to identify factors related to sarcopenia and their association with prospective falls in patients with rheumatoid arthritis between 24 and 85 years of age.

## Methods and materials

### Study design and sample

The present study is a prospective, observational study. A random sample of German patients with rheumatoid arthritis were recruited at the Department of Rheumatology and Clinical Immunology and the Centre of Muscle and Bone Research of the Charité – Universitätsmedizin Berlin. Subjects were included if they were at least 18 years old, had a confirmed diagnosis of rheumatoid arthritis according to the 2010 American College of Rheumatology classification criteria [22], were able to walk with or without walking aid and signed a written informed consent form. The exclusion criteria were (1) injury affecting muscle function during the last 3 months, (2) acute illness or exacerbation of a chronic disease affecting muscle function, (3) pregnancy, and (4) contraindications for x-ray exposure. The study was conducted according to the Declaration of Helsinki, and required ethical approval by the ethics committee of Charité-Universitätsmedizin Berlin (EA4/155/16) and by the German Radiation Protection Office (Z 5–2246/2–2016-145).

To address the patient and public involvement (PPI) recommended by the European League Against Rheumatism (EULAR) [23], two patient representatives were involved throughout the research process. After 2 days of training organised by the Deutsche Rheuma-Liga Bundesverband e.V., they provided input on the research questions and study process from the patients’ perspective, commented on the results and supported the dissemination among their peers.

**Fig. 1:**
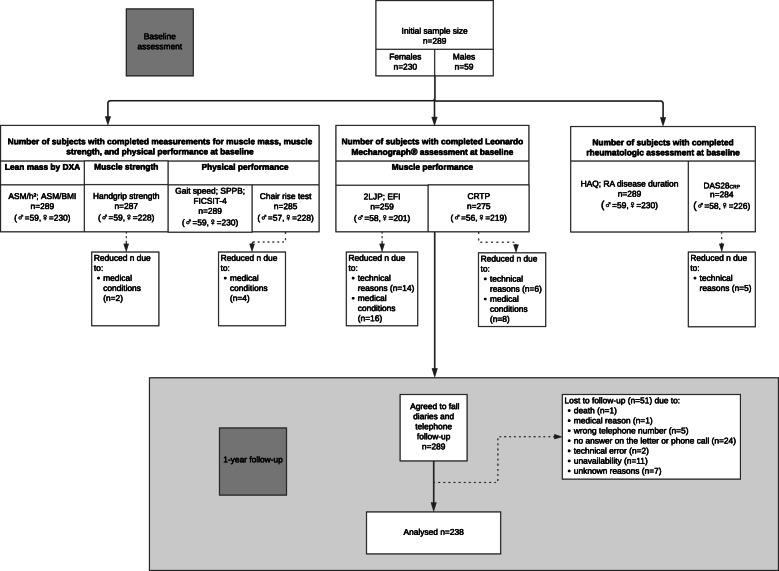
Flowchart of the study

The study on prospective falls was attached to a cross-sectional study investigating the prevalence of sarcopenia in RA (SarkoRA). The sample size calculation assumed a prevalence of 25% in RA patients with a two-sided 95% confidence interval. A sample size of *n* = 289 was calculated based on the sample size estimation software nQuery + nTerim 3.0. A total of 289 subjects participated in the baseline assessment, which was performed between 2017 and 2019. The flowchart presents the numbers of subjects with available data by assessment instrument (Fig. 1). The largest portion of incomplete data was due to technical errors on the Leonardo Mechanograph® during muscle power assessment of two-leg jumps (2LJP) and chair rise tests (CRTP).

All of the 289 persons participating in the baseline assessment agreed to the one-year follow-up. The subjects were followed for 1 year using quarterly fall diaries to report recent falls. Participants were contacted by telephone to specify the reported falls in terms of their character or to remind them to return the diaries. 238 subjects (82%) completed the entire 1-year observation and were included in the present analysis. The majority of the non-responders did not send back their fall diaries and did not answer the reminding telephone calls or letters (*n* = 24). Other reasons for drop-out were death (*n* = 1) and medical condition (*n =* 1). Further, wrong contact information (*n* = 5), technical reasons (*n =* 2) or unavailability (*n* = 11) led to a reduced sample size for follow-up (Fig. 1).

### Fall assessment

Falls were defined as “an unexpected event in which the participants come to rest on the ground, floor, or lower level” as recommended in the consensus statement of the ProFaNE group [24]. Falls due to syncopes or accidents with external forces were excluded. Participants were categorized into two groups according to their fall diaries: the fall group with one or more falls and the non-faller group with no falls during the one-year observation.

Previous falls have been reported as a risk factor for falls, both in the elderly [25] and in patients with RA [6, 26]. So, fall history within the 6 months before the baseline examination was assessed. The participants were asked “Did you fall during the past 6 months? (Yes/No)”. The dichotomous variable “previous falls” was integrated in the analysis.

### Anthropometry

Anthropometric informations such as height and weight were measured in underwear without shoes by a digital measuring and weighing station (Seca 764). Body mass index (BMI) was calculated as body weight (kg)/height (m^2^).

### Assessment of disease

Relevant clinical parameters for rheumatoid arthritis such as RA disease duration, C-reactive protein (CRP) and the Disease Activity Score (DAS28_CRP_) [27] were collected.

### Questionnaire

To assess functional status and disability the participants were interviewed with the Health Assessment Questionnaire (HAQ) [28]. This is a widely used instrument to assess 8 dimensions of daily life activities. The HAQ is rated on a scale of 0 to 3, where 0 represents no impairments and 3 severely limited. The instrument shows high associations with fear of falling [29] and falls [12, 30, 31].

General physical activity was assessed with the self-constructed question “Are you physically active regularly?” Physical activity is a relevant preventative factor for sarcopenia [32]. A dichotomous variable “physical activity” with the answers yes/no was integrated in the analysis.

### Muscle mass

Lean mass was measured using Dual X-Ray-Absorptiometry (DXA) (Lunar iDXA, GE Medical Systems, Madison, Wisconsin, USA; EnCore Software Version 16.1) as a surrogate parameter for muscle mass following standard operating procedures according to the GE Lunar manual. Appendicular skeletal muscle mass (ASM) was calculated based on the summation of the lean mass of the arms and legs [33, 34]. For the analysis, ASM was adjusted for height squared (ASM/h^2^ in kg/m^2^) and body mass index (ASM/BMI in m^2^) [10].

### Muscle strength

Maximum handgrip strength was measured in a seated position, with the elbow flexed 90° and placed on the armrest. The wrists were hanging freely and were in a neutral position. The subjects were asked to squeeze the digital hand dynamometer Leonardo Mechanograph GF® (Novotec Medical GmbH, Pforzheim, Germany, Software BAS v4.4) as tightly as possible. The test included one trial per hand and the best was included into the analysis [35].

### Physical performance

#### Gait speed

To evaluate gait speed, the participants were asked to walk 6.45 m at a normal pace along a quiet corridor. Subjects were allowed to use their walking aids. The time was recorded by a stopwatch and gait speed in m/s was calculated for the analysis.

#### Short Physical Performance Battery

Functional performance was evaluated by the Short Physical Performance Battery (SPPB), scoring the results of the balance assessment (Romberg, Semitandem, Tandem), gait speed, and chair rise test. The SPPB is a widely used instrument with high reliability and validity to assess functional performance of lower extremity [36] and to predict falls in older patients [37–40]. The maximum possible total score is 12 indicating high functional performance. In addition, a categorial variable was generated along validated cut-point of less than or equal to 6 points (poor performer), 7 to 9 points (moderate performer) and higher than 9 points (good performer) [40, 41].

#### FICSIT-4

Another performance test used in the study was the FICSIT-4 [42]. The assessment evaluates the standing balance of Romberg, Semitandem, Tandem and one-leg stance by using a composite score. The scale ranges from 0 to 5 points reflecting whether a position could be held for 10 s or not. 5 points are the maximum possible score with holding all positions for 10 s. The FICSIT-4 is integrated as a nominal variable as well as a dichotomous variable: “low FICSIT-4” with the score of 1–4 categorised as ‘poor balance’ and a score of 5 as the reference group with ‘good balance’.

### Sarcopenia assessment

To examine sarcopenic changes in the study population, variables were generated based on established sarcopenia definitions and cut-off values. According to the sarcopenia definition by the European Working Group on Sarcopenia in Older People (EWGSOP2), relevant outcome measures were low handgrip strength, high chair rise time, low muscle mass, low physical performance and low gait speed [10]. The EWGSOP2 recommends an algorithm to identify and confirm sarcopenia. First, to discover “probable sarcopenia” low muscle strength is defined by grip strength < 27 kg in men and < 16 kg in women, and/or chair rise time higher than 15 s. Next, muscle mass with ASM/h^2^ lower than 7 kg/m^2^ in men and lower than 5.5 kg/m^2^ in woman “confirms sarcopenia”. “Severe sarcopenia” is determined by a reduced gait speed of less than or equal to 0.8 m/s, low SPPB score ≤ 8, Timed-up-and-go-time of ≥20 s or a 400 m walk time of six minutes or more (or not completing the test) in individuals with confirmed sarcopenia [10]. In the recent study gait speed was integrated into the algorithm.

For the sarcopenia assessment, dichotomous variables were generated to classify the results of the study participants into low or high along the cut-off values (yes/no). Those subjects unable to perform one of the assessments such as the chair rise test or grip strength were categorised in the lowest category.

Based on the definition of The Foundation for the National Institutes of Health (FNIH) a second sarcopenia assessment was included in the analysis [43]. The FNIH recommends cut-off values for ASM/BMI in men lower than 0.789 m^2^ and women lower than 0.512 m^2^ in combination with low grip strength (men < 26 kg, women < 16 kg).

### Leonardo mechanography

The chair rise test (CRT) and jumping mechanography were carried out on the Leonardo Mechanograph® Ground Reaction Force Plate (Novotec Medical GmbH, Pforzheim, Germany, software package 4.4). For all measurements, a frequency of 800 Hz was used. During the assessment, subjects were allowed to wear their own flat shoes and clothing.

#### Two-leg countermovement jump

Initially, the participants performed three two-leg jumps (2LJ) as countermovement jumps on the platform. For further analysis, the jump with the highest maximum total relative power per body weight in W/kg during lift off (2LJP_rel_) was selected. Based on this variable, the Leonardo software calculated the Esslinger Fitness Index (EFI in %), which represents a percentage of an age- and sex-specific reference value.

#### Chair rise test

For the CRT, a bench with a height of 45 cm was installed on the Leonardo force plate. The subjects were instructed to rise and sit down 5 times as quick as possible with their arms folded in front of their chest. The time was measured with a stopwatch (CRT in sec). The Leonardo force plate recorded the maximum total relative power per body weight in W/kg during the rise phases (CRTP_rel_).

### Statistical analysis

Descriptive data were generated for all variables and are presented by means and standard deviation (SD) for continuous and normally distributed variables, and median and interquartile ranges for skewed variables. The summary of binary and categorical variables is presented by using frequencies and percentages. The study participants were categorised as a “non-responder”, a “non-faller” or as a “faller”, if they fell at least once during the observational period. Differences between the group of non-fallers and fallers were evaluated using independent sample t-tests, Mann-Whitney-U-tests or Chi-squared tests as appropriate.

Logistic regression analysis was used to explore associations between physical performance, disease-specific parameters, and muscle mass with falling. First, univariate logistic regression was performed followed by a multiple logistic regression with adjustment for age and sex. Then, all variables that were found to be significant in the univariate analyses or considered as possible risk factors were included in a multiple logistic regression analysis. Results are presented as odds ratios (OR), 95% confidence intervals and *p-*values. To compare the logistic regression models, the − 2 log-likelihood, Cox-Snell R^2^ and Nagelkerke’s R^2^ are reported.

Receiver-operating characteristics (ROC) were used to compare the predictive value of the measurements for falls. The area under the curve (AUC) was used to determine the quality of the prediction and was reported with its 95% confidence interval and *p-*value. A larger and significant AUC refers to a better predictive ability. However, ROC analysis did not reveal specific cut-off values for any of the variables due to poor performance in determining between fall and no fall cases, hence results are not shown. Significance levels were set at the 5% level. All analyses were carried out using SPSS, version 27.

## Results

### Incidence of falls

Of the 238 subjects who completed the follow-up (82.3%), 48 (20.2%) persons reported at least one fall during the one-year follow-up, 83.3% of those were females.

### Characteristics of study sample

Table 1 presents demographic and baseline characteristics of non-fallers, fallers and non-responders. The mean age of the responder sample (non-fallers and fallers, *n* = 238) was 60.2 (± 11.6) years (not shown). The responders were significant older than the non-responders (56.9 ± 9.4 years, *p* = 0.036, not shown) and had higher baseline physical performance according to the EFI (82% vs. 76%, *p* = 0.034, not shown). In other demographic and baseline disease-specific parameters, no significant differences were found between responders and non-responders.

**Table 1:** Characteristics and results of the baseline assessment by follow-up status of participants

Variables	Responder ***n*** = 238					Non-responder ***n*** = 51	
	Non-Fallers		Fallers		***p-***value^**1**^		
	n	mean (± SD)	n	mean (± SD)		n	mean (± SD)
**Demographics**							
Age (years)	190	59.2 (± 11.9)	48	63.7 (± 9.5)	**0.016**	51	56.9 (± 9.4)
-Females	147	58.4 (± 11.9)	40	63.6 (± 9.9)	**0.013**	43	56.7 (± 9.7)
Females, n, (%)	190	147 (77.4)	48	40 (83.3)	0.368	51	43 (84.3)
Height (m)	190	1.67 (± 0.08)	48	1.64 (± 0.07)	**0.017**	51	1.66 (± 0.08)
Weight (kg)	190	77.3 (± 14.0)	48	73.1 (± 12.0)	0.059	51	75.3 (± 15.0)
BMI (kg/m^2^)	190	27.1 (± 4.5)	48	26.5 (± 4.0)	0.401	51	26.9 (± 4.9)
Previous falls (yes), n, (%)	190	25 (13.2)	48	11 (22.9)	0.092	51	14 (27.5)
Physical activity (yes), n, (%)	190	137 (72.1)	48	36 (75.0)	0.688	51	26 (51.0)
**Medical data**							
RA disease duration (y)^2^	190	9.0 (4.0–16.0)	48	11.0 (5.0–19.7)	0.33	51	10.0 (6.0–19.0)
DAS28_CRP_ (score)^2^	189	2.07 (1.62–2.85)	45	2.33 (1.69–3.25)	0.161	50	2.35 (1.64–3.03)
-Low disease activity ≤3.2, n, (%)		154 (81.1)		33 (66.8)	0.229		39 (76.5)
-Moderate disease activity 3.2 ≤ 5.1, n, (%)		34 (17.9)		12 (25.0)			9 (17.6)
-High disease activity > 5.1, n, (%)		1 (0.5)		0			2 (3.9)
HAQ (score)^2^	190	0.37 (0.0–1.12)	48	0.81 (0.41–1.5)	**0.001**	51	0.75 (0.0–1.5)
**Physical performance tests**							
Handgrip strength (kg)	189	24.8 (± 9.0)	48	22.45 (± 8.2)	0.102	50	22.97 (± 8.1)
Gait speed (m/s)	190	1.24 (± 0.21)	48	1.23 (± 0.25)	0.70	51	1.19 (± 0.2)
CRT (sec)	187	9.43 (± 3.2)	47	9.69 (±3.6)	0.635	51	10.32 (± 3.8)
SPPB (total score)^2^	190	11.0 (11.0–12.0)	48	11.0 (10.0–12.0)	0.645	51	11.0 (10.0–12.0)
SPPB (score category)							
-Poor performer: 0–6, n, (%)		7 (3.7)		1 (2.1)	0.710		3 (5.9)
-Moderate performer: 7–9, n, (%)		20 (10.5)		7 (14.6)			7 (13.7)
-Good performer: 10–12, n, (%)		163 (85.8)		40 (83.3)			41 (80.4)
FICSIT-4 (score)^2^	190	5.0 (0.0)	48	5.0 (4.0–5.0)	**0.030**	51	5.0 (0.0)
Low FICSIT-4 (1–4), n, (%)	190	28 (14.7)	48	14 (29.2)	**0.019**	51	10 (19.6)
**Lean mass DXA**							
ASM/h^2^ (kg/m^2^)	190	6.89 (± 1.04)	48	6.64 (± 0.9)	0.13	51	6.73 (± 1.0)
ASM/BMI (m^2^)	190	0.73 (± 0.1)	48	0.69 (± 0.1)	**0.04**	51	0.70 (± 0.1)
**Leonardo tests**							
CRTP_rel_ (W/kg)	182	10.35 (± 2.8)	44	9.73 (± 2.5)	0.18	49	9.45 (± 2.0)
2LJP_rel_ (W/kg)	169	27.19 (± 6.9)	45	25.32 (± 5.3)	**0.05**	45	25.60 (± 6.8)
EFI (%)	169	81.20 (± 17.3)	45	83.67 (± 16.0)	0.38	45	75.74 (± 17.3)

Notes

^1^ *p-*value of unpaired t-test or Mann-Whitney-U-Test or Chi^2^-test, bold values significant difference between fallers and non-fallers.

^2^ Data are presented as median (interquartile range).

Regarding medical data, the fall group appeared to have a longer RA disease duration with a median of 11 years (range, 1 to 46 years) and a higher Disease Activity Score with a median of 2.33 points compared to non-fallers (median of 9 years and 2.07 points, respectively). There was no statistically significant difference between both groups with regard to the RA disease duration (*p* = 0.33) and DAS28_CRP_ (*p* = 0.161). The majority of both groups had low disease activity at baseline according DAS28_CRP_ (*n* = 187, 66%). Significant differences were found between fallers and non-fallers regarding age, height, and HAQ score, i.e. fallers were significant older (*p* = 0.016) and more restricted in the activities of daily life (*p* = 0.001) (Table 1).

### Sarcopenia assessment

Table 2 presents variables related to relevant sarcopenia definitions with numbers of subjects and percentages. Based upon the criteria of EWGSOP2 [10] 4.6% of the responders were classified as having sarcopenia. According to the FNIH Sarcopenia Project definition [43], 2.9% of the responders were identified as sarcopenic. No significant differences were found between the groups of fallers and non-fallers.

**Table 2:** Sarcopenia assessment of responder sample (*n =* 238)

Variables	All responders		Non-Fallers		Fallers		***p-***value
	**n**	**n (%)**	**n**	**n (%)**	**n**	**n (%)**	
Low handgrip strength, n, (%)^a^	238	47 (19.7)	190	36 (18.9)	48	11 (22.9)	0.537
Low gait speed, n, (%)^a^	238	11 (4.6)	190	7 (3.7)	48	4 (8.3)	0.17
High CRT time, n, (%)^a^	238	17 (7.1)	190	14 (7.4)	48	3 (6.3)	0.788
Low muscle mass (ASM/h^2^), n, (%)^a^	238	30 (12.6)	190	25 (13.2)	48	5 (10.4)	0.609
Low muscle mass (ASM/BMI), n, (%)^b^	238	15 (6.3)	190	10 (5.3)	48	5 (10.4)	0.189
Sarcopenia (EWGSOP2)^a^							
No sarcopenia	238	180 (75.6)	190	145 (76.3)	48	35 (72.9)	0.593
Probable sarcopenia	238	47 (19.7)	190	37 (19.5)	48	10 (20.8)	
Confirmed sarcopenia	238	11 (4.6)	190	8 (4.2)	48	3 (6.3)	
Severe sarcopenia	238	0	190	0	48	0	
Sarcopenia (FNIH)^b^							
No sarcopenia	238	231 (97.1)	190	185 (97.4)	48	46 (95.8)	0.574
Sarcopenia	238	7 (2.9)	190	5 (2.6)	48	2 (4.2)	

Notes

^a^according to cut-off-values by EWGSOP2: low grip strength: men < 27 kg, women < 16 kg; low gait speed < 0.8 m/s; high CRT time > 15 s; low muscle mass ASM/h^2^: men < 7 kg, women < 5,5 kg; sarcopenia classification: probable: low grip strength and/or high CRT time; confirmed: low grip strength and/or high CRT time and low ASM/h^2^; severe: low grip strength and/or high CRT time and low ASM/h^2^ and low gait speed

^b^according to cut-off-values by FNIH: low muscle mass ASM/BMI: men< 0.789m^2^, women< 0.512m^2^ and low grip strength: men< 26 kg, women< 16 kg.

### Physical performance, muscle mass and mechanography

In the assessment of physical performance, muscle mass and Leonardo mechanography patients with a fall status had lower values in grip strength, gait speed, ASM/h^2^ and ASM/BMI, CRTP and 2LJP than non-fallers. However, the only significant group differences were found in ASM/BMI (*p* = 0.04) and 2LJP (*p* = 0.05). The fall group showed a numerically longer time in chair rising, but this was not significant compared to non-fallers (*p* = 0.635). The assessment of FICSIT-4, revealed that the fall group has a significant greater range in holding the balance positions for ten seconds (IQR 4.0–5.0; *p* = 0.03) and a significantly higher proportion of fallers (29.2%) had a low FICSIT-4 score (1–4) (*p* = 0.019) (Table 1).

### Factors associated with prospective falls

A significant association between age and prospective falls was found (OR = 1.04; CI 1.01–1.07, *p* = 0.017). There was no significant influence of sex on fall risk (*p* = 0.523) (Table 3). Sarcopenia was not associated with future falls (*p* = 0.689; *p* = 0.870) (Table 4).

**Table 3:** Unadjusted and adjusted associations for fall risk

	Unadjusted						Adjusted for age and sex^**1**^								
Predictors	OR	[95% CI]	p-value	AUC	[95% CI]	p-value	OR	[95% CI]	p-value	-2 Log-Likelihood	Cox- Snell R²	Nagelkerke's R²	AUC	[95% CI]	p-value
**Medical data**															
Age	**1.04**	[1.01-1.07]	**0.017**	0.60	[0.52-0.69]	0.026	n.a.								
Sex^1^	1.46	[0.64-3.36]	0.370	0.47	[0.38-0.56]	0.523	n.a.								
HAQ	**1.62**	[1.1-2.38]	**0.014**	0.65	[0.57-0.73]	0.002	1.52	[1.01-2.27]	**0.043**	215.25	0.05	0.07	0.65	[0.56-0.74]	0.001
DAS28_CRP_	1.28	[0.93-1.76]	0.125	0.57	[0.47-0.66]	0.161	1.35	[0.97-1.87]	0.072	218.65	0.04	0.07	0.64	[0.55-0.72]	0.004
RA disease duration	1.02	[0.98-1.05]	0.281	0.55	[0.45-0.64]	0.331	1.01	[0.97-1.04]	0.769	223.93	0.03	0.05	0.61	[0.53-0.7	0.014
**Physical performance tests**															
Handgrip strength (kg)	0.97	[0.93-1.0]	0.104	0.4	[0.33-0.5]	0.063	0.98	[0.94-1.03]	0.423	230.73	0.03	0.05	0.63	[0.54-0.72]	0.006
Gait speed (m/s)	0.75	[0.18-3.17]	0.694	0.5	[0.38-0.56]	0.543	1.67	[036-7.83]	0.514	231.33	0.03	0.05	0.61	[0.53-0.7]	0.016
CRT (sec)	1.02	[0.93-1.12]	0.634	0.5	[0.43-0.62]	0.602	0.98	[0.88-1.09]	0.698	225.03	0.04	0.06	0.63	[0.54-0.72]	0.007
SPPB (score, metric)	0.97	[0.80-1.17]	0.736	0.5	[0.39-0.57]	0.666	1.10	[0.89-1.35]	0.394	231.01	0.03	0.05	0.62	[0.53-0.7]	0.014
SPPB (score, category)															
Poor performer: 0-6	0.58	[0.07-4.87]	0.618	0.49	[0.40-0.58]	0.819	0.25	[0.03-2.25]	0.214	229.72	0.04	0.06	0.63	[0.53-0.72]	0.007
Moderate performer: 7-9	1.43	[0.56-3.61]	0.453				1.00	[0.38-2.65]	0.997						
Good performer: 10-12	Reference						Reference								
FICSIT-4 (score)															
Score 1-4	**2.38**	[1.13-5.0]	**0.022**	0.43	[0.33-0.52]	0.123	1.65	[0.72-3.8]	0.239	230.41	0.04	0.06	0.62	[0.54-0.71]	0.008
Score 5	Reference						Reference								
FICSIT-4 (metric)	0.74	[0.50-1.1]	0.137	0.43	[0.34-0.53]	0.150	0.94	[0.60-1.46]	0.768	231.67	0.03	0.05	0.61	[0.53-0.70]	0.015
**Lean mass DXA**															
ASM/h² (kg/m²)	0.8	[0.57-1.08]	0.135	0.4	[0.35-0.52]	0.170	0.82	[0.56-1.21]	0.314	230.73	0.04	0.06	0.62	[0.53-0.71]	0.009
ASM/BMI (m²)	0.10	[0.01-1.29]	0.078	0.4	[0.35-0.53]	0.208	0.18	[0.0-7.13]	0.359	230.90	0.03	0.05	0.61	[0.52-0.7]	0.019
**Leonardo tests**															
CRTP_rel_ (W/kg)	0.9	[0.81-1.04]	0.183	0.4	[0.35-0.54]	0.241	1	[0.87-1.14]	0.965	212.52	0.04	0.07	0.6	[0.55-0.73]	0.004
2LJP_rel_ (W/kg)	1	[0.90-1.0]	0.093	0.4	[0.34-0.52]	0.146	1.00	[0.94-1.07]	0.974	210.43	0.04	0.07	0.63	[0.54-0.72]	0.009
EFI (%)²	n.a.						1	[0.99-1.03]	0.387	210.30	0.04	0.07	0.5	[0.44-0.63]	0.438

^1^Male = Reference

^2^EFI is already adjusted for age and sex

**Table 4:** Unadjusted and adjusted associations between risk factors of sarcopenia and fall risk

	Unadjusted						Adjusted for age and sex*^3^								
Predictors	OR	[95% CI]	p-value	AUC	[95% CI]	p-value	OR	[95% CI]	p-value	-2 Log-Likelihood	Cox- Snell R²	Nagelkerke's R²	AUC	(95% CI)	p-value
Low handgrip strength^1^*	1.3	[0.59-2.73]	0.538	0.5	[0.43-0.61]	0.671	1.16	[0.53-2.52]	0.714	232.99	0.03	0.04	0.61	[0.52-0.69]	0.021
Low gait speed^1^	2.38	[0.67-8.48]	0.182	0.52	[0.43-0.62]	0.619	1.27	[0.32-5.04]	0.732	231.65	0.03	0.05	0.62	[0.53-0.71]	0.009
High CRT time^1^	0.84	[0.23-3.04]	0.788	0.49	[0.40-0.58]	0.905	0.53	[0.14-2.06]	0.364	230.85	0.03	0.05	0.62	[0.53-0.71]	0.012
Low muscle mass (ASM/h²)^1^*	0.77	[0.28-2.12]	0.610	0.49	[0.40-0.58]	0.769	0.74	[0.26-2.07]	0.566	232.78	0.03	0.04	0.61	[0.52-0.69]	0.023
Low muscle mass (ASM/BMI)²*	2.09	[0.68-6.44]	0.198	0.53	[0.43-0.62]	0.581	1.85	[0.59-5.75]	0.290	232.07	0.03	0.05	0.61	[0.52-0.70]	0.018
Sarcopenia (EWGSOP2)^1^															
No sarcopenia	Reference			0.52	[0.43-0.61]	0.689	Reference			231.23	0.03	0.05	0.62	[0.53-0.71]	0.01
Probable sarcopenia	1.12	[0.51-2.47]	0.779				0.92	[0.40-2.08]	0.838						
Confirmed sarcopenia	1.55	[0.39-6.16]	0.531				1.64	[0.40-6.61]	0.489						
Sarcopenia (FNIH)²*															
No sarcopenia	Reference			0.51	[0.41-0.60]	0.87	Reference			232.95	0.03	0.04	0.6	[0.52-0.69]	0.027
Sarcopenia	1.61	[0.30-8.56]	0.577				1.44	[0.27-7.74]	0.673						

^1^according to cut-off-values by EWGSOP2: low grip strength: men <27 kg, women <16 kg; low gait speed <0.8m/s; high CRT time >15s; low muscle mass ASM/h²: men <7 kg, women <5,5 kg;

sarcopenia classification: probable: low grip strength and/or high CRT time; confirmed: low grip strength and/or high CRT time and low ASM/h²; severe: low grip strength and/or high CRT time and low ASM/h² and low gait speed

² according to cut-off-values by FNIH: low muscle mass ASM/BMI: men<0.789m², women<0.512m² and low grip strength: men<26kg, women<16kg

*low grip strength and low muscle mass are already adjusted for sex

³ Male = Reference

HAQ was found to be an independent risk factor for falling. The univariate logistic regression analysis of HAQ showed a strong and significant association with prospective falls. For each 1-point increase of the score the probability of falling rose by 1.62 (CI 1.1–2.38, *p* = 0.014). Unadjusted HAQ had the highest independent predictive value of the study parameters to identify fallers (AUC = 0.65; CI 0.57–0.73, *p* = 0.002) (Table 3).

The univariate logistic regression showed that participants with a low score in FICSIT-4 had a higher fall risk compared to those with a high score (OR = 2.38; CI 1.13–5.0, *p* = 0.022). The adjusted logistic regression detected a higher and significant AUC (0.62; CI 0.54–0.71, *p* = 0.008), thus a better predictive quality for falls (Table 3).

For all other physical performance tests, DXA measure, Leonardo assessment, and health questionnaires no association with falls could be found (Table 3).

Table 5 shows the results of the multiple logistic regression analysis. The analysis confirms that age (OR 1.06; CI 1.01–1.10, *p =* 0.008) and HAQ (OR 1.89; CI 1.00–3.56, *p* = 0.048) are associated with falls. SPPB (OR 1.57; CI 1.13–2.18) was found to be significant in the multiple regression model (*p* = 0.007).

**Table 5:** Multiple logistic regression analysis

	Multiple logistic regression analysis		
Predictors	OR	[95% CI]	p-value
**Medical data**			
Age	**1.06**	[1.01-1.10]	**0.008**
Sex^1^	1.15	[0.35-3.82]	0.821
HAQ	**1.89**	[1.00-3.56]	**0.048**
DAS28_CRP_	1.37	[0.93-2.02]	0.114
RA disease duration	1.00	[0.96-1.04]	0.927
Previous falls (yes)	1.87	[0.75-4.66]	0.179
**Physical performance tests**			
Handgrip strength (kg)	1.02	[0.96-1.08]	0.591
SPPB (score, metric)	**1.57**	[1.13-2.18]	**0.007**
FICSIT-4 (score)			
Score 1-4	2.21	[0.75-6.49]	0.147
Score 5	Reference		
**Lean mass DXA**			
ASM/h² (kg/m²)	0.73	[0.47-1.13]	0.160

The results of the multiple logistic regression analysis are shown with the adjusted odds ratios (OR) and 95% confidence intervals (CI)

^1^Male = Reference

## Discussion

The present prospective study was designed to examine the associations between variables of sarcopenia and physical performance with falls in a cohort of patients with rheumatoid arthritis. The results of this study indicate that age, HAQ, and low score of FICSIT-4 are independently associated with prospective falls. No association of sarcopenia and falls was found. This study is the first to report prospective data on sarcopenia and falls in a rheumatic sample. Further, there is no other study using the FICSIT-4 assessment in a rheumatic sample to date. The Leonardo Mechanograph® used in the study is also an innovative measurement device that provides promising data on muscle mechanography and for which there are limited studies published in the context of rheumatoid arthritis.

In this cohort, one in five participants with rheumatoid arthritis reported at least one fall during the 1-year observational period (20.2%). This is a relatively low rate compared to other prospective studies, which indicated an incidence rate up to 50% [4, 6, 7]. Only two other prospective studies reported lower incidence rates of 10.1% [12] and 18.3% [44]. Further retrospective studies described incidence rates between 27 and 54% [13, 14, 30, 45–48], which are notably higher than the results of this study. However, retrospective studies are known to be more imprecise [49]. Other reasons for the comparatively low incidence rate in the present results may involve the low RA disease activity in 78% of the patients and the relatively young age (half were under 60 years old).

Nevertheless, age was an independent risk factor for falls in this study. The majority of studies with RA patients have found no association of age and falls [5, 13, 45, 50]. This is surprising, given the fact that cohorts of healthy subjects showed a strong association between the parameters age and falls [51]; thus, age is widely accepted as a risk factor for falls from the age of 65 years onwards [52]. To our knowledge, only two other studies reporting on RA patients identified age as significant risk factor for falls [44, 53].

Likewise, the existing literature on sex as a risk factor for falls is inconsistent. In the present study sex was not found to be a significant risk factor for falls. One possible confounder might be the relatively small portion of male patients (21.4%) in this study, as the sample consisted mainly of females. However, this accurately reflects the sex distribution of rheumatoid arthritis in the general population, affecting more women than men. A systematic review of Brenton-Rule et al. [5] also reported no associations of sex with falls in a population of RA patients. In contrast, the longitudinal study by Mamoto et al. [44] confirmed female sex as relevant risk factor for falls in patients with RA, which is consistent with study results in healthy populations. Falls are more common in females with an increased risk of fractures, but males have a higher fall-associated mortality [52]. As falls are a multi-causal phenomenon, it is not only sex to determine the risk, but additional sex- and age-related physiological deterioration or RA disease-associated processes.

Using age and sex as covariates in the adjusted logistic regression models, the results showed a reduced risk of falls in most models, although the AUCs increased and were significant, thus indicating that they are possibly able to discriminate between falls and no falls. Possible reasons for the corresponding variables are explained below.

A systematic review and meta-analysis analysing the association between sarcopenia and falls in older adults by Yeung et al. [54] reported inconsistent results in a total of 22 studies. 45% of the analysed studies reported significant associations and the others did not. No difference was found whether a prospective or cross-sectional study design was used. Results differed depending on the underlying sarcopenia definition. By use of the EWGSOP1 definition (14 studies) sarcopenia was significantly associated with falls, by use of the FNIH definition (2 studies) not. It has been shown that the EWGSOP1 definition reveals a higher prevalence of sarcopenia than the EWGSOP2 definition [55, 56], so comparability with this study is limited. Despite the use of different sarcopenia definitions and cut-off values in the present study, the proportion of sarcopenic patients was probably too low to show associations with falls. However, because the incidence of falls is higher in RA patients with sarcopenia compared to the general population [11], it is important to be aware of these factors.

Sarcopenia can be confirmed by a combination of low muscle strength and muscle mass [10, 43]. The group comparisons of this study revealed lower values for grip strength, ASM/h^2^ and ASM/BMI in the fall group. For ASM/BMI, the difference between fallers and non-fallers was even significant. However, using the cut-off values for muscle mass, handgrip strength and physical performance according the sarcopenia definitions [10, 43] the findings of the current study do not support an association with falls in the logistic regression analysis. There is conflicting evidence for those physiological risk factors. A study by Stanmore et al. revealed a significant association between falls and lower limb muscle strength measured using the chair rise test [15]. This is supported by recent retrospective studies [57, 58], yet cannot be confirmed by the results of the present study. In contrast, handgrip strength of subjects with RA was not associated with falls in any study independent of the study design [7, 48, 53, 59]. In patients with a chronic inflammatory arthritis several factors may affect handgrip strength measurement, including pain, stiffness or deformities [60]. As the majority of the patients in our study cohort presented low disease activity [61], it can be assumed that active inflammatory processes did not have a substantial impact on grip strength and is therefore not associated with falls. Although handgrip strength is supposed to be correlated with total body muscle strength [62], the results of this study suggest that assessing muscle strength of the lower extremity is better than the measurement of upper limb strength for the evaluation of the risk of falls in patients with RA.

Due to the chronically elevated concentration of inflammatory cytokines that promote proteolysis in patients with RA and reduced physical activity, these individuals are at higher risk for sarcopenia, even at a younger age [11, 63, 64]. As such, it seems reasonable that longer RA disease duration and higher disease activity were previously associated with sarcopenic changes [11]. Since this was a population with low RA disease activity, it might not be surprising that the association with disease activity was not demonstrated. The RA disease duration has been described by numerous studies as a non-significant factor for falls [6, 7, 12, 50]. This is also supported by the present study results showing no association with falls and duration of disease or disease activity (DAS28_CRP_). It remains to be considered that the DAS28_CRP_ primarily examines the disease activity of the upper extremity. In the lower extremity, only the knee joints were assessed. It is therefore not possible to evaluate the influence of damaged or inflamed joints of the feet or hip on postural control. Böhler et al. [45] stated that RA disease activity should be measured at the moment of the fall, as this can change during the period of a prospective study. Since the assessment was conducted at the beginning of the study in our sample and the majority of subjects had low disease activity at that time, no associations with falls could be found. According to Zonzini Gaino et al. [47], an analysis of individuals with a predominantly high RA disease activity or a separate analysis for recurrent fallers may provide stronger associations of disease activity and falls. An interesting aspect would have been the repetition of the baseline assessment at the end of the one-year follow-up.

In the present study, fallers with RA were more restricted in activities of daily life than non-fallers. The HAQ score had the strongest association with falls. This observation supports previous prospective research which identified self-reported physical activity and mobility as risk factors for falls [12, 15]. Furthermore, retrospective studies confirmed the association of HAQ with falls [13, 46]. A retrospective study by Armstrong et al. [30] demonstrated that two of eight domains (walking and chair rising) were associated with fall history. Activities of daily living like walking, dressing or reaching require muscle strength, power and balance. Reduced physical capacity could contribute to impaired physical performance in activities of daily living, thus predisposing people to falls.

Previous studies have demonstrated that poor balance is associated with an increased risk of falling in both healthy subjects [65, 66] and patients with RA [7, 15]. According to a systematic review by Brenton-Rule et al. [5], balance can be assessed by various balance tests such as standing balance, four balance test, one-leg standing time, postural sway or composite scores such as the Tinetti Balance Test. In the present study, the composite score of FICSIT-4 was used including standing time and four standing positions. Group comparisons between fallers and non-fallers showed significant differences in ‘FICSIT-4’ and ‘low FICSIT-4’ score. ‘Low FICSIT-4’ score showed a significant association with falls in the univariate logistic regression analysis, but not in a multiple regression analysis. RA seems to have a negative impact on postural control. This effect is increased in subjects with old age and high BMI [67]. Several factors seem to affect balance measures in patients with RA, such as foot deformities, pain, loss of muscle strength and impaired mobility [68]. Moreover, dizziness due to medication side effects may influence balance assessment [15]. Chronic inflammation processes and the side effects of drugs are able to impair sensory input and neuromuscular responses, leading to reduced balance and increased risk of falls [69]. To our knowledge there is no other study using the FICSIT-4 score in a study population with RA to date. The results suggest that the FICSIT-4 score is a promising alternative to other established balance assessments.

Physical performance tests such as SPPB, including gait speed and chair rise test did not reveal significant differences between fallers and non-fallers and were also not significant predictors for falls in the univariate analyses. These results reflect those of Lourenco et al. [50], who also found no prospective association of SPPB with falls in patients with RA. Gait speed alone was also a superior predictor to SPPB in the cross-sectional study by Dietzel et al. [53]. This finding is contrary to previous studies in samples of healthy elderly people which have suggested that SPPB is appropriate to assess fall risk [40]. However, a retrospective study in a sample of patients with ankylosing spondylitis observed higher fall risk in patients with SPPB scores ≤6 [39]. In a recent retrospective study by Kawabata et al. [70] chair rise time and balance assessment appeared more appropriate than the total SPPB score for predicting falls. This is in accordance with the results of the current study, showing that the FICSIT-4 as a balance assessment was able to discriminate between RA patients with and without falls in a better way. In contrast, the multiple regression analysis showed that a combination of age, self-reported physical activity and mobility by the HAQ and the evaluation of physical performance using the SPPB was the strongest predictor of falls.

Contrary to expectations, this study did not find that muscle power variables could predict future falls in the logistic regression models. Muscle power is considered a key variable in terms of physical performance and activities of daily living. Compared to muscle strength and muscle mass, it decreases earlier and gets more pronounced over the lifespan [71]. Low muscle power seems to be a relevant risk factor for falls and the impairment of activities of daily life in healthy samples over a wide range of ages [16, 17]. In patients with RA, an association with falls has not yet been proven [53]. It is important to note that in the present study, fallers showed lower values in muscle power of CRT and quite significant for 2LJ. Therefore, muscle power indicated group differences between fallers and non-fallers, but was not able to predict prospective falls in the logistic regression models. However, although the AUCs of the adjusted logistic regression models were significant, indicating that they are likely to be able to discriminate between falls and no falls, the ORs were not significant. This is in line with Dionyssiotis et al. [19] reporting on cross-sectional group comparisons between women with rheumatic diseases, osteoporotic postmenopausal women, healthy postmenopausal women and healthy premenopausal women. The research team found a significant decline of all kinematic parameters in women with rheumatic diseases compared to the other groups. Possible reasons were the RA disease inherent processes and the side effects of medication used [19]. Also, Dietzel et al. [53] investigated muscle power in older individuals with RA compared to healthy people in a cross-sectional study. They identified significant group differences with decreased muscle power compared to the healthy group, but found no associations with falling. Further prospective studies, which take muscle power in samples of patients with RA into account, will need to be undertaken.

### Limitations

There are some limitations in the present study. The results were affected by the relatively low rate of falls during the observational period. Most of the prospective studies showed higher incidence rates between 36 and 50% [6, 7, 15]. One reason for this may be that the study was initially powered for the prevalence of sarcopenia in RA patients. The majority of the sample had low disease activity and half of the patients were under 60 years of age. Although the sample consisted of subjects of all ages, the study included mainly participants who were physically able to visit the outpatient clinic of a university facility in an urban area. This may also have led to an underestimation of patients with fear of falling, a fall history and patients at high risk of falling did not participate in the study. However, the group comparison between fallers and non-fallers showed no significant difference concerning previous falls as well as the multiple regression model was not significant for fall history. Furthermore, it is possible that patients have already participated in a prevention programme for falls or underwent physiotherapy due to their chronic disease or a history of falls. As the initial objective of this study was to evaluate the prevalence of sarcopenia in RA, physical activity in relation to fall prevention was not assessed differentially. The variable “physical activity” covers self-reported activity, but not whether it is sport, specific training or everyday activity.

The strengths of the current study include the prospective one-year study design, the use of quarterly fall diaries and a low drop-out rate. Nevertheless, the non-responder sample often showed poor results in the baseline assessment. Therefore, it is possible that the subjects with high fall risk were lost to follow-up.

As the original study focused on the prevalence of sarcopenia in RA, medication [5], comorbidities [48] and foot deformities [13, 72] which are known to increase fall risk were not documented in the study. In particular, foot deformities in patients with RA have a major impact on balance, muscle activity, and strength of the lower limb. As mentioned earlier, each disease management should include the improvement of the flexibility of the joints and muscles of the feet and increasing proprioception of the foot for a better postural control.

For the performance of balance, the time-of-day affect according to Bouchaala et al. [73] plays a crucial role. In their study of women with RA balance performance and risk of falls fluctuate during the day and was higher in the morning and in the evening compared to 2 pm. The researchers reported the influence of stiffness, pain, swelling and limited range of motion on functional capacities and balance performance. The time-of-day effect was not considered in this study and the subjects were assessed throughout the day for reasons of study logistics.

## Conclusion

The purpose of the current study was to determine associations of variables of sarcopenia and physical performance with falls in a sample of German patients with RA. Sarcopenia according to two different consensus definitions was not associated with prospective falls. The results of this study indicate that age, HAQ, and low FICSIT-4 score are independently associated with prospective falls.

Patients with RA are at a higher risk of falls than healthy subjects. The incidence of fractures and other fall-related injuries is more pronounced in patients with RA. Therefore, a fall risk assessment focussing on age, self-reported physical activity and mobility and physical performance in patients with RA should be integrated in all health-care routines. The present study suggests that the FICSIT-4 score as well as the established SPPB are promising tools to assess fall-risk in patients with RA. Future research should examine whether interventions that improve mobility and proprioception of the foot and the prevention of joint deformities are able to reduce fall risk in this patient population.

## Data Availability

The datasets used and/or analysed during the current study are available from the corresponding author on reasonable request.

## Abbreviations

ASM: Appendicular skeletal muscle mass
AUC: Area under the curve
CRT: Chair rise test
CRTP: Muscle power of chair rise test
EFI: Esslinger Fitness Index (%)
HAQ: Health Assessment Questionnaire
RA: Rheumatoid Arthritis
2LJ: Two-leg jumps
2LJP: Muscle power of two-leg jumps

## References

[1] Safiri S, Kolahi AA, Hoy D, Smith E, Bettampadi D, Mansournia MA, Almasi-Hashiani A, Ashrafi-Asgarabad A, Moradi-Lakeh M, Qorbani M, Collins G, Woolf AD, March L, Cross M (2019). Global, regional and national burden of rheumatoid arthritis 1990−2017: a systematic analysis of the global burden of disease study 2017. Ann Rheum Dis.

[2] Steffen AHJ, Goffrier B, Bätzing J (2017). Epidemiologie der rheumatoiden Arthritis in Deutschland – eine Analyse anhand bundesweiter vertragsärztlicher Abrechnungsdaten.

[3] Yesim A, Ulus Y, Berna T, Tomak L, Zahiroglu Y, Bilgici A, et al. AB0353 associated factors for falls and fear of falling in ambulatory patients with rheumatoid arthritis: a comparative study with healthy subjects. Ann Rheum Dis. 2017. 10.1136/annrheumdis-2017-eular.2888.

[4] Stanmore EK, Oldham J, Skelton DA, O'Neill T, Pilling M, Campbell AJ, Todd C (2013). Fall incidence and outcomes of falls in a prospective study of adults with rheumatoid arthritis. Arthritis Care Res.

[5] Brenton-Rule A, Dalbeth N, Bassett S, Menz HB, Rome K (2015). The incidence and risk factors for falls in adults with rheumatoid arthritis: a systematic review. Semin Arthritis Rheum.

[6] Smulders E, Schreven C, Weerdesteyn V, van den Hoogen FH, Laan R, Van Lankveld W (2009). Fall incidence and fall risk factors in people with rheumatoid arthritis. Ann Rheum Dis.

[7] Hayashibara M, Hagino H, Katagiri H, Okano T, Okada J, Teshima R (2010). Incidence and risk factors of falling in ambulatory patients with rheumatoid arthritis: a prospective 1-year study. Osteoporos Int.

[8] Organization WH (2008). WHO global report on falls prevention in older age.

[9] Haagsma JA, Olij BF, Majdan M, van Beeck EF, Vos T, Castle CD, Dingels ZV, Fox JT, Hamilton EB, Liu Z, Roberts NLS, Sylte DO, Aremu O, Bärnighausen TW, Borzì AM, Briggs AM, Carrero JJ, Cooper C, el-Khatib Z, Ellingsen CL, Fereshtehnejad SM, Filip I, Fischer F, Haro JM, Jonas JB, Kiadaliri AA, Koyanagi A, Lunevicius R, Meretoja TJ, Mohammed S, Pathak A, Radfar A, Rawaf S, Rawaf DL, Riera LS, Shiue I, Vasankari TJ, James SL, Polinder S (2020). Falls in older aged adults in 22 European countries: incidence, mortality and burden of disease from 1990 to 2017. Inj Prev.

[10] Cruz-Jentoft AJ, Bahat G, Bauer J, Boirie Y, Bruyère O, Cederholm T, Cooper C, Landi F, Rolland Y, Sayer AA, Schneider SM, Sieber CC, Topinkova E, Vandewoude M, Visser M, Zamboni M, Bautmans I, Baeyens JP, Cesari M, Cherubini A, Kanis J, Maggio M, Martin F, Michel JP, Pitkala K, Reginster JY, Rizzoli R, Sánchez-Rodríguez D, Schols J, Writing Group for the European Working Group on Sarcopenia in Older People 2 (EWGSOP2), and the Extended Group for EWGSOP2 (2018). Sarcopenia: revised European consensus on definition and diagnosis. Age Ageing.

[11] Torii M, Hashimoto M, Hanai A, Fujii T, Furu M, Ito H, Uozumi R, Hamaguchi M, Terao C, Yamamoto W, Uda M, Nin K, Morita S, Arai H, Mimori T (2019). Prevalence and factors associated with sarcopenia in patients with rheumatoid arthritis. Mod Rheumatol.

[12] Furuya T, Yamagiwa K, Ikai T, Inoue E, Taniguchi A, Momohara S, Yamanaka H (2009). Associated factors for falls and fear of falling in Japanese patients with rheumatoid arthritis. Clin Rheumatol.

[13] Mikos M, Kucharska E, Lulek AM, Kłosiński M, Batko B. Evaluation of risk factors for falls in patients with rheumatoid arthritis. Med Sci Monit. 2020;26. 10.12659/msm.921862. 10.12659/MSM.921862 PMC7177037 32292180

[14] Metli NB, Kurtaran A, Akyüz M (2014). AB0307 impaired balance and fall risk in rheumatoid arthritis patients. Ann Rheum Dis.

[15] Stanmore EK, Oldham J, Skelton DA, O'Neill T, Pilling M, Campbell AJ, Todd C (2013). Risk factors for falls in adults with rheumatoid arthritis: a prospective study. Arthritis Care Res.

[16] Dietzel R, Felsenberg D, Armbrecht G (2015). Mechanography performance tests and their association with sarcopenia, falls and impairment in the activities of daily living - a pilot cross-sectional study in 293 older adults. J Musculoskelet Neuronal Interact.

[17] Parsons CM, Edwards MH, Cooper C, Dennison EM, Ward KA (2020). Are jumping mechanography assessed muscle force and power, and traditional physical capability measures associated with falls in older adults? Results from the Hertfordshire cohort study. J Musculoskelet Neuronal Interact.

[18] Dionyssiotis Y, Galanos A, Michas G, Trovas G, Lyritis GP (2010). Assessment of musculoskeletal system in women with jumping mechanography. Int J Women's Health.

[19] Dionyssiotis Y, Skarantavos G, Kantaidou I, Papadatou MC, Papagelopoulos P, Angoules A, et al. Evaluation of physical performance in musculoskeletal and rheumatic diseases with jumping mechanography. J Frailty Sarcopenia Falls. 2019. 10.22540/jfsf-04-116. 10.22540/JFSF-04-116 PMC7155307 32300726

[20] van Staa TP, Geusens P, Bijlsma JW, Leufkens HG, Cooper C (2006). Clinical assessment of the long-term risk of fracture in patients with rheumatoid arthritis. Arthritis Rheum.

[21] Huusko TM, Korpela M, Karppi P, Avikainen V, Kautiainen H, Sulkava R (2001). Threefold increased risk of hip fractures with rheumatoid arthritis in Central Finland. Ann Rheum Dis.

[22] Aletaha D, Neogi T, Silman AJ, Funovits J, Felson DT, Bingham CO (2010). 2010 rheumatoid arthritis classification criteria: an American College of Rheumatology/European league against rheumatism collaborative initiative. Arthritis Rheum.

[23] de Wit MP, Berlo SE, Aanerud GJ, Aletaha D, Bijlsma JW, Croucher L (2011). European league against rheumatism recommendations for the inclusion of patient representatives in scientific projects. Ann Rheum Dis.

[24] Lamb SE, Jørstad-Stein EC, Hauer K, Becker C (2005). Development of a common outcome data set for fall injury prevention trials: the prevention of falls network Europe consensus. J Am Geriatr Soc.

[25] Tinetti ME, Speechley M, Ginter SF (1988). Risk factors for falls among elderly persons living in the community. N Engl J Med.

[26] Bugdayci D, Paker N, Rezvani A, Kesiktas N, Yilmaz O, Sahin M, Ince N (2013). Frequency and predictors for falls in the ambulatory patients with rheumatoid arthritis: a longitudinal prospective study. Rheumatol Int.

[27] Mäkinen H, Kautiainen H, Hannonen P, Möttönen T, Korpela M, Leirisalo-Repo M, Luukkainen R, Puolakka K, Karjalainen A, Sokka T (2007). Disease activity score 28 as an instrument to measure disease activity in patients with early rheumatoid arthritis. J Rheumatol.

[28] Bruce B, Fries JF (2005). The health assessment questionnaire (HAQ). Clin Exp Rheumatol.

[29] Akyol Y, Ulus Y, Tander B, Tomak L, Zahiroğlu Y, Bilgici A, Kuru Ö (2018). Falls, fear of falling, and associated factors in ambulatory patients with rheumatoid arthritis: a comparative study with healthy controls. Turkish J Physical Med Rehabilitation.

[30] Armstrong C, Swarbrick CM, Pye SR, O'Neill TW (2005). Occurrence and risk factors for falls in rheumatoid arthritis. Ann Rheum Dis.

[31] Marques WV, Cruz VA, Rego J, da Silva NA (2014). The influence of physical function on the risk of falls among adults with rheumatoid arthritis. Rev Bras Reumatol.

[32] Steffl M, Bohannon RW, Sontakova L, Tufano JJ, Shiells K, Holmerova I (2017). Relationship between sarcopenia and physical activity in older people: a systematic review and meta-analysis. Clin Interv Aging.

[33] Baumgartner RN, Koehler KM, Gallagher D, Romero L, Heymsfield SB, Ross RR, Garry PJ, Lindeman RD (1998). Epidemiology of sarcopenia among the elderly in New Mexico. Am J Epidemiol.

[34] Heymsfield SB, Smith R, Aulet M, Bensen B, Lichtman S, Wang J, Pierson RN (1990). Appendicular skeletal muscle mass: measurement by dual-photon absorptiometry. Am J Clin Nutr.

[35] Kennedy D, Jerosch-Herold C, Hickson M. The reliability of one vs. Three Trials of Pain-free Grip Strength in Subjects with Rheumatoid Arthritis. J Hand Ther. 2010. 10.1016/j.jht.2010.05.002. 10.1016/j.jht.2010.05.002 20971419

[36] Guralnik JM, Simonsick EM, Ferrucci L, Glynn RJ, Berkman LF, Blazer DG, Scherr PA, Wallace RB (1994). A short physical performance battery assessing lower extremity function: association with self-reported disability and prediction of mortality and nursing home admission. J Gerontol.

[37] Quadri P, Tettamanti M, Bernasconi S, Trento F, Loew F (2005). Lower limb function as predictor of falls and loss of mobility with social repercussions one year after discharge among elderly inpatients. Aging Clin Exp Res.

[38] Hars M, Audet MC, Herrmann F, De Chassey J, Rizzoli R, Reny JL (2018). Functional performances on admission predict in-hospital falls, injurious falls, and fractures in older patients: a prospective study. J Bone Miner Res.

[39] Dursun N, Sarkaya S, Ozdolap S, Dursun E, Zateri C, Altan L, Birtane M, Akgun K, Revzani A, Aktas İ, Tastekin N, Celiker R (2015). Risk of falls in patients with ankylosing spondylitis. J Clin Rheumatol.

[40] Veronese N, Bolzetta F, Toffanello ED, Zambon S, De Rui M, Perissinotto E (2014). Association between short physical performance battery and falls in older people: the Progetto Veneto Anziani study. Rejuvenation Res.

[41] Guralnik JM, Ferrucci L, Pieper CF, Leveille SG, Markides KS, Ostir GV, Studenski S, Berkman LF, Wallace RB (2000). Lower extremity function and subsequent disability: consistency across studies, predictive models, and value of gait speed alone compared with the short physical performance battery. J Gerontol A Biol Sci Med Sci.

[42] Rossiter-Fornoff JE, Wolf SL, Wolfson LI, Buchner DM. A cross-sectional validation study of the FICSIT common data base static balance measures. Frailty and Injuries: Cooperative Studies of Intervention Techniques. J Gerontol A Biol Sci Med Sci. 1995. 10.1093/gerona/50a.6.m291. 10.1093/gerona/50a.6.m291 7583799

[43] Studenski SA, Peters KW, Alley DE, Cawthon PM, McLean RR, Harris TB (2014). The FNIH sarcopenia project: rationale, study description, conference recommendations, and final estimates. J Gerontol A Biol Sci Med Sci.

[44] Mamoto K, Inui K, Okano T, Sugioka Y, Tada M, Koike T, Nakamura H (2017). Incidence rate of falls and its risk factors in patients with rheumatoid arthritis compared to controls: four years of the TOMORROW study. Mod Rheumatol.

[45] Böhler C, Radner H, Ernst M, Binder A, Stamm T, Aletaha D, et al. Rheumatoid arthritis and falls: the influence of disease activity. Rheumatology (Oxford, England). 2012. 10.1093/rheumatology/kes198. 10.1093/rheumatology/kes198 22879462

[46] Kaz Kaz H, Johnson D, Kerry S, Chinappen U, Tweed K, Patel S. Fall-related risk factors and osteoporosis in women with rheumatoid arthritis. Rheumatology (Oxford, England). 2004. 10.1093/rheumatology/keh304. 10.1093/rheumatology/keh304 15252210

[47] Fessel KD, Nevitt MC (1997). Correlates of fear of falling and activity limitation among persons with rheumatoid arthritis. Arthritis Care Res.

[48] Jamison M, Neuberger GB, Miller PA (2003). Correlates of falls and fear of falling among adults with rheumatoid arthritis. Arthritis Rheum.

[49] Ganz DA, Higashi T, Rubenstein LZ (2005). Monitoring falls in cohort studies of community-dwelling older people: effect of the recall interval. J Am Geriatr Soc.

[50] Lourenço MA, Carli F, de Assis MR. Characterization of falls in adults with established rheumatoid arthritis and associated factors. Adv Rheumatol (London, England). 2018. 10.1186/s42358-018-0021-0. 10.1186/s42358-018-0021-0 30657096

[51] Deandrea S, Lucenteforte E, Bravi F, Foschi R, La Vecchia C, Negri E (2010). Risk factors for falls in community-dwelling older people: a systematic review and Meta-analysis. Epidemiology..

[52] WHO (2007). WHO Global report on Falls Prevention in Older Age.

[53] Dietzel R, Felsenberg D, Armbrecht G. AB0355 muscle power and fall risk in rheumatoid arthritis. Ann Rheum Dis. 2017. 10.1136/annrheumdis-2017-eular.1957.

[54] Yeung SSY, Reijnierse EM, Pham VK, Trappenburg MC, Lim WK, Meskers CGM, Maier AB (2019). Sarcopenia and its association with falls and fractures in older adults: A systematic review and meta-analysis. J Cachexia Sarcopenia Muscle.

[55] de Freitas MM, de Oliveira VLP, Grassi T, Valduga K, Miller MEP, Schuchmann RA, Souza KLA, de Azevedo MJ, Viana LV, de Paula TP (2020). Difference in sarcopenia prevalence and associated factors according to 2010 and 2018 European consensus (EWGSOP) in elderly patients with type 2 diabetes mellitus. Exp Gerontol.

[56] Reiss J, Iglseder B, Alzner R, Mayr-Pirker B, Pirich C, Kässmann H, Kreutzer M, Dovjak P, Reiter R (2019). Consequences of applying the new EWGSOP2 guideline instead of the former EWGSOP guideline for sarcopenia case finding in older patients. Age Ageing.

[57] Delialioglu SU, Kamaci GK, Ozel S, Yurdakul F, Bodur H. SAT0130 evaluation of the factors related with risk of falling and fear of falling in patients with rheumatoid arthritis. Ann Rheum Dis. 2017. 10.1136/annrheumdis-2017-eular.2022.

[58] Zonzini Gaino J, Barros Bértolo M, Silva Nunes C, de Morais BC, Sachetto Z, Davitt M (2019). Disease-related outcomes influence prevalence of falls in people with rheumatoid arthritis. Ann Phys Rehabil Med.

[59] Duyur Cakit BNB, Erdem HR, Karagöz A, Saracoglu M. Fear of Falling, Fall Risk and Disability in Patients with Rheumatoid Arthritis. Turk J Rheumatol. 2011. 10.5606/tjr.2011.034.

[60] Beenakker KG, Ling CH, Meskers CG, de Craen AJ, Stijnen T, Westendorp RG (2010). Patterns of muscle strength loss with age in the general population and patients with a chronic inflammatory state. Ageing Res Rev.

[61] Tada M, Yamada Y, Mandai K, Hidaka N (2019). Correlation between frailty and disease activity in patients with rheumatoid arthritis: data from the CHIKARA study. Geriatr Gerontol Int.

[62] Rantanen T, Era P, Heikkinen E (1994). Maximal isometric strength and mobility among 75-year-old men and women. Age Ageing.

[63] Targowski T (2017). Sarcopaenia and rheumatoid arthritis. Reumatologia..

[64] Müller R, Kull M, Põlluste K, Valner A, Lember M, Kallikorm R. Factors Associated With Low Lean Mass in Early Rheumatoid Arthritis: A Cross- Sectional Study. Medicina (Kaunas, Lithuania). 2019. 10.3390/medicina55110730. 10.3390/medicina55110730 PMC6915666 31717450

[65] Pizzigalli L, Micheletti Cremasco M, Mulasso A, Rainoldi A (2016). The contribution of postural balance analysis in older adult fallers: a narrative review. J Bodyw Mov Ther.

[66] Johansson J, Nordström A, Gustafson Y, Westling G, Nordström P (2017). Increased postural sway during quiet stance as a risk factor for prospective falls in community-dwelling elderly individuals. Age Ageing.

[67] Aydoğ E, Bal A, Aydoğ ST, Cakci A (2006). Evaluation of dynamic postural balance using the Biodex stability system in rheumatoid arthritis patients. Clin Rheumatol.

[68] Silva KN, Mizusaki Imoto A, Almeida GJ, Atallah AN, Peccin MS, Fernandes Moça Trevisani V. Balance training (proprioceptive training) for patients with rheumatoid arthritis. Cochrane Database Syst Rev. 2010. 10.1002/14651858.CD007648.pub2. 10.1002/14651858.CD007648.pub2 20464755

[69] Ramos-Remus C, Duran-Barragan S, Castillo-Ortiz JD (2012). Beyond the joints: neurological involvement in rheumatoid arthritis. Clin Rheumatol.

[70] Kawabata K, Matsumoto T, Kasai T, Chang SH, Hirose J, Tanaka S (2020). Association between fall history and performance-based physical function and postural sway in patients with rheumatoid arthritis. Mod Rheumatol.

[71] Wiegmann S, Felsenberg D, Armbrecht G, Dietzel R (2021). Longitudinal changes in muscle power compared to muscle strength and mass. J Musculoskelet Neuronal Interact.

[72] Brenton-Rule A, Dalbeth N, Menz HB, Bassett S, Rome K (2017). Are foot and ankle characteristics associated with falls in people with rheumatoid arthritis? A prospective study. Arthritis Care Res.

[73] Bouchaala F, Laatar R, Lahiani M, Zouabi A, Borji R, Rebai H, Sahli S (2020). Time of day effect on balance performance, functional capacities and risk of fall in women with rheumatoid arthritis. Chronobiol Int.

